# Specific Recognition of Influenza A/H1N1/2009 Antibodies in Human Serum: A Simple Virus-Free ELISA Method

**DOI:** 10.1371/journal.pone.0010176

**Published:** 2010-04-14

**Authors:** Mario M. Alvarez, Felipe López-Pacheco, José M. Aguilar-Yañez, Roberto Portillo-Lara, Gonzalo I. Mendoza-Ochoa, Sergio García-Echauri, Pamela Freiden, Stacey Schultz-Cherry, Manuel I. Zertuche-Guerra, David Bulnes-Abundis, Johari Salgado-Gallegos, Leticia Elizondo-Montemayor, Martín Hernández-Torre

**Affiliations:** 1 Centro de Biotecnología-FEMSA, Tecnológico de Monterrey, Monterrey, New León, México; 2 Department of Infectious Disease, St. Jude Children's Research Hospital, Memphis, Tennessee, United States of America; Louisiana State University, United States of America

## Abstract

**Background:**

Although it has been estimated that pandemic Influenza A H1N1/2009 has infected millions of people from April to October 2009, a more precise figure requires a worldwide large-scale diagnosis of the presence of Influenza A/H1N1/2009 antibodies within the population. Assays typically used to estimate antibody titers (hemagglutination inhibition and microneutralization) would require the use of the virus, which would seriously limit broad implementation.

**Methodology/Principal Findings:**

An ELISA method to evaluate the presence and relative concentration of specific Influenza A/H1N1/2009 antibodies in human serum samples is presented. The method is based on the use of a histidine-tagged recombinant fragment of the globular region of the hemagglutinin (HA) of the Influenza A H1N1/2009 virus expressed in *E. coli*.

**Conclusions/Significance:**

The ELISA method consistently discerns between Inf A H1N1 infected and non-infected subjects, particularly after the third week of infection/exposure. Since it does not require the use of viral particles, it can be easily and quickly implemented in any basic laboratory. In addition, in a scenario of insufficient vaccine availability, the use of this ELISA could be useful to determine if a person has some level of specific antibodies against the virus and presumably at least partial protection.

## Introduction

Seasonal influenza causes thousands of deaths annually, worldwide. In the United States alone, more than 50,000 patients die yearly due to influenza-like illness and its consequences [1]. Influenza continues to be a disease that causes major suffering and economic loss to modern societies [2]–[6].

On April 26^th^ 2009, an epidemiological emergency related to an Influenza A H1N1virus was declared in México [7], [8]. During the first few weeks of surveillance, the virus spread worldwide to 30 countries (as of May 11) by human-to-human transmission. By June 2009, only a few weeks later, Influenza A/H1N1/2009 was declared a Level VI Pandemic by the World Health Organization (WHO) [8], [9]. This represented the first time in history that a disease had been designated at this risk level.

The total number of people infected around the world is difficult to estimate, and the diagnosis efforts have been surpassed by the advance of the disease. It is believed that, in the period between April-December 2009, millions of people were infected. By the end of the year 2009, WHO had declared that 12,000 persons had died due to Influenza A/H1N1; half of them in North America (México, Canada and USA). On the other hand, a significant fraction of the world population may already have been exposed to the virus (between 12 [10] and 40% [11], [12]) and, although asymptomatic, could be at least partially immune to the disease [10]–[12].

A more precise assessment of the total number of persons that have been infected would require the large scale use of techniques to specifically determine the presence and relative concentration of 2009 H1N1 influenza virus antibodies in serum samples. Besides its obvious epidemiological significance, the availability of these types of techniques would also allow the rapid discernment of potentially immune subjects among those more susceptible to infection in a given population. This is particularly relevant in a situation where there is insufficient availability of vaccine, such as the one that was experienced in most developing countries, including México, the epicenter of this pandemic.

**Figure 1:**
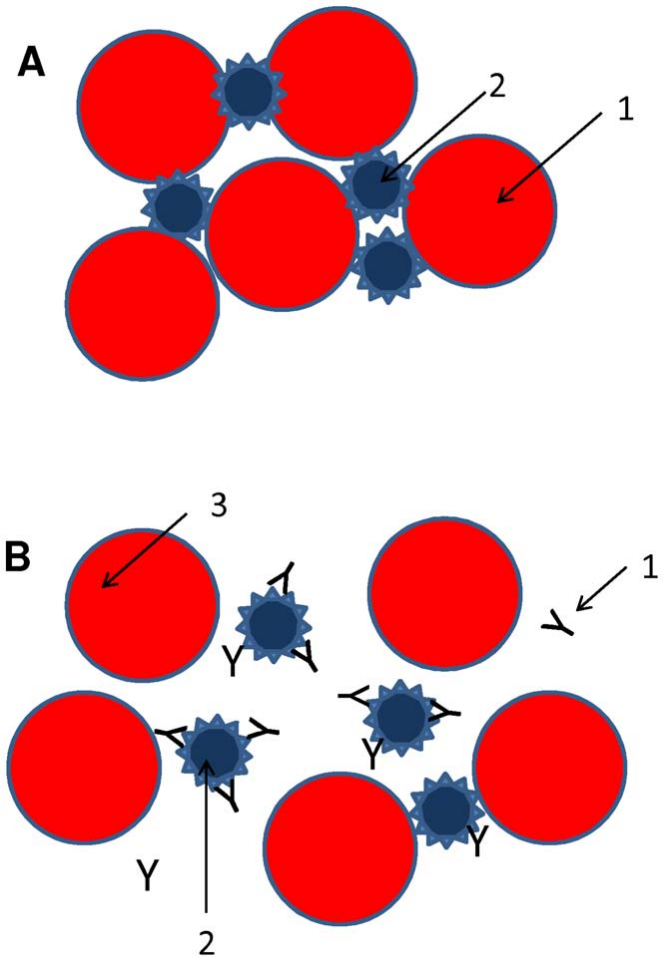
Hemagglutination inhibition assay. (A) Schematic representation of hemagglutination using Influenza viral particles. In the absence of agglutinationon inhibitors, the hemagglutinin from viral capsids (1) agglutinates chicken, turkey or human erythrocytes(2). (B) Schematic representation of hemagglutination inhibition. In the presence of neutralizing antibodies (1) that specifically recognize the hemagglutinin from a influenza virus (2), the process of hemagglutination is inhibited proportionally to the concentration and binding affinity of the neutralizing antibodies.

Typically, titration of anti-influenza antibodies is based on experiments that evaluate the ability of a serum sample to inhibit hemagglutination (Figure 1). Hemagglutinin is the most external and most antigenic surface protein of influenza viruses. As its name suggests, it induces hemagglutination by specifically binding erythrocyte surface receptors (Figure 1a). At a fixed viral concentration, and in the presence of an HA binding molecule (e.g., neutralizing antibodies from a convalescent influenza patient), the observed hemagglutination inhibition should be proportional to the concentration of the inhibitor (Figure 1b). This is the basic concept behind the hemagglutination inhibition (HI) assay [13].

HI has become a relatively universal protocol for estimating antibody titers against a particular influenza strain. The lowest dilution of a viral suspension at which serum samples still inhibit agglutination is determined by visual inspection and indicates neutralization titer. Recent literature includes a number of examples of the application of HI assays in the context of epidemiological or clinical studies [10]–[12], [14], [15], in vaccine development [16], [17], in analytical development [18], or in more fundamental studies. However, HI assays are not infallible for some applications and they posses several important limitations.

Although HI titers have been strongly recommended [19] and are commonly used to predict and qualify vaccine efficacy, the use of titers often fails when live attenuated vaccines are evaluated, because the assay does not encompass all of the immune mediators of protection [18]. Strictly speaking, HI titers only indicate interference of some anti-HA antibodies with the sialidated receptors at the erythrocyte membrane. Consequently, adequate performance of the titer test also depends on the nature and quality of the erythrocytes used [20]. More importantly, for practical implementation, HI assays require the use of viruses. In the context of epidemiological studies, the analysis of hundreds to thousands of samples is typical [10]–[12], [14], [21]. To conduct these studies using standard HI assays demands an immense supply of virus, which in turn requires the implementation of virus propagation protocols and involvement of specially trained personnel. In addition, particularly in the case of highly infective or new influenza strains, level II or III bio-safety laboratory spaces and protocols would also be mandatory for safe implementation of virus culture.

Recently, a number of alternatives to HI have been investigated. One of these is the microneutralization method, which has also increased in use for evaluating the titer of neutralizing antibodies [14], [15], [21], [22]. However, microneutralization methods also rely on the use of the actual virus. In addition, its standardization for a particular influenza strain is not a trivial procedure [22]. Several immunological-based methods have also been proposed to qualitatively or quantitatively evaluate the presence of anti-influenza antibodies in animal or human serum samples [23], [24], [25], [26].

In the present paper, we document the development and use of an ELISA method that will specifically recognize 2009 H1N1 influenza virus antibodies in human serum samples, based on use of a recombinant fragment of the globular region of the protein hemagglutinin (HA) of the 2009 H1N1 virus. This protein, produced in *E. coli*, specifically and effectively recognizes antibodies from serum obtained from positive H1N1 virus-infected patients, as diagnosed by standard PCR protocols [27], in samples taken as soon as two weeks after infection. In addition, this antigen can be massively produced and easily purified by standard methodologies, providing the possibility of large scale supply sufficient for large epidemiological studies.

The immunoassay proposed here shows significant advantages over conventional HI assays; primarily: (a) it does not require fresh chicken or turkey erythrocytes (which are limited in their commercial availability); (b) it does not require virus manipulation (and therefore does not require special infrastructure or safety protocols); and (c) interpretation of results rests on absorbance readings, instead of subjective visual estimations of agglutination.

## Results and Discussion

### Rationale of the assay

The structures of HA antigenic sites vary not only among different subtypes of viruses but also within the same subtype. The continuous antigenic drifts and occasional antigenic shifts that arise from this continuous evolutionary variation enable human influenza viruses to escape the human immune system [28]. Neutralizing antibodies are generally regarded as not broadly cross-reactive among HA subtypes. Indeed, influenza A viruses subtypes (e.g., H1, H2, etc) are defined as serotypes that are determined by neutralization or hemagglutination inhibition (HI) tests using polyclonal antisera to the respective HA subtypes, which have little cross-reactivity to the other HA subtypes. To our knowledge, there is only one report [29] of a monoclonal antibody, directed to a well conserved conformational epitope in influenza A viruses, that is capable of recognizing several different HA serotypes (H1, H2, H3, H5, H9, H13).

Circulating H1N1/2009 influenza virus is antigenically distinct from seasonal human A(H1N1) [30] and other seasonal influenza A viruses [21]. Therefore, conceptually, an ELISA method could be used to conclusively discriminate between subjects previously exposed to 2009 H1N1 influenza virus and those not exposed. If we accept that these immunogenic differences between the 2009 H1N1 influenza virus and other previously or actually circulating strains exist, then a sufficiently specific immunological test should also discriminate between subjects exposed to 2009 H1N1 influenza virus and other influenza A viruses. With this type of assay, we also avoid the use of viral particles by using a recombinant protein, the HA fragment of the 2009 H1N1 influenza virus (specific details will be provided later) produced in *E. coli* as the antigen of an ELISA method. *E. coli* was chosen as expression host due to the simplicity of producing large quantities (of the order of 1 to 3 g/L) of recombinant proteins in a short time period using standard bacterial culture techniques. We found that this fragment of HA can be specifically recognized by antibodies directed against the 2009 H1N1 influenza virus.

From a more fundamental perspective, one of the most important contributions of this work is the conclusive demonstration that a recombinant antigen from bacterial origin (and therefore not glycosylated) can be specifically recognized by human antibodies targeted against a specific influenza strain. Glycosylation is normally viewed as playing a role in antigen-antibody recognition. However, for influenza virus, the mandatory requirement of glycosylation for adequate antigenic biorecognition has not been demonstrated [31]–[33].

Five antigenic sites, all located in the globular region of hemagglutinin, have been conclusively identified in influenza A H1N1viruses [29], [34]–[39]. Some of them are in the vicinity but not associated with glycosylation sites [39]–[41]. Indeed, all of them have been reported to be individually accessible to specific antibodies [29], [34], [36], [38], [39].

In addition, reports [42], [43] from Song et al. and Chiu et al. have demonstrated that fractions of HA expressed in *E. coli*, and therefore non-glycosylated, are immunogenic in animal models. These HA fragments corresponded to the globular region, particularly to the HA1 domain.

**Figure 2:**
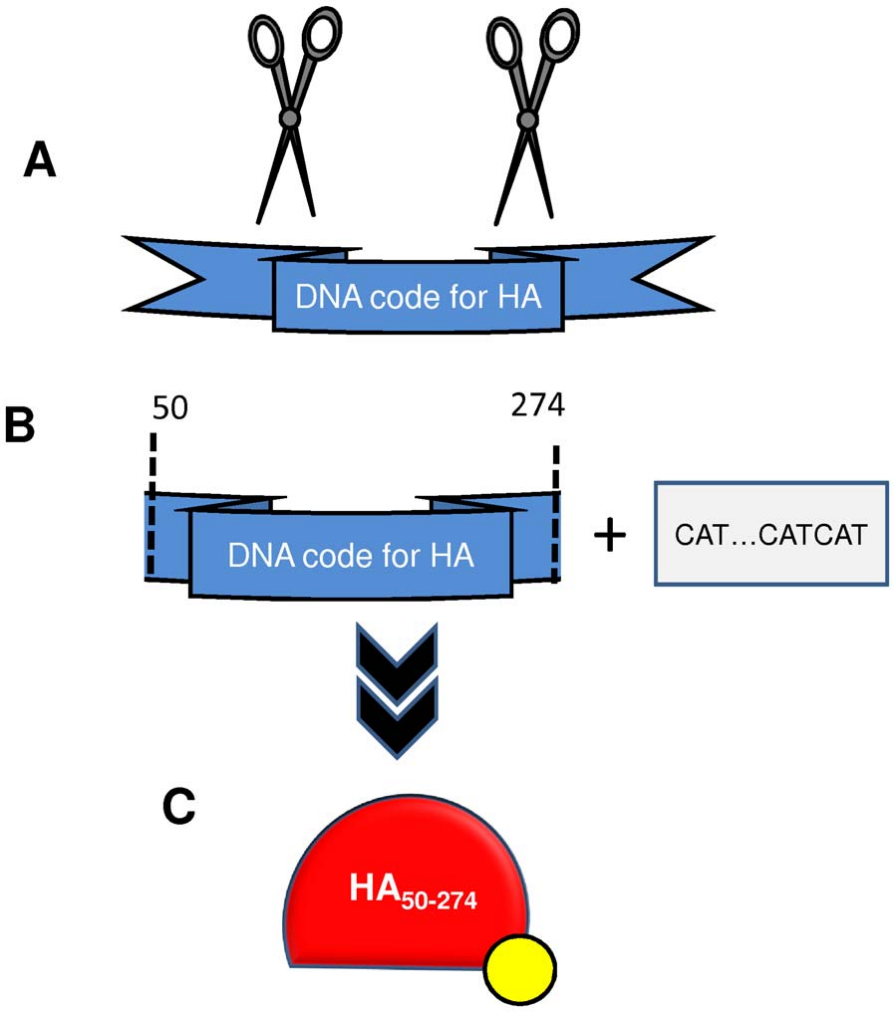
Strategy used to express protein HA_50–274_-H1N1 in *E. coli*. The DNA region specifically encoding for the HA amino-acid sequence between residues 50 and 274 was preceded by a promoter region and added in the N-terminus with a sequence encoding for a histydine tag.

**Figure 3:**
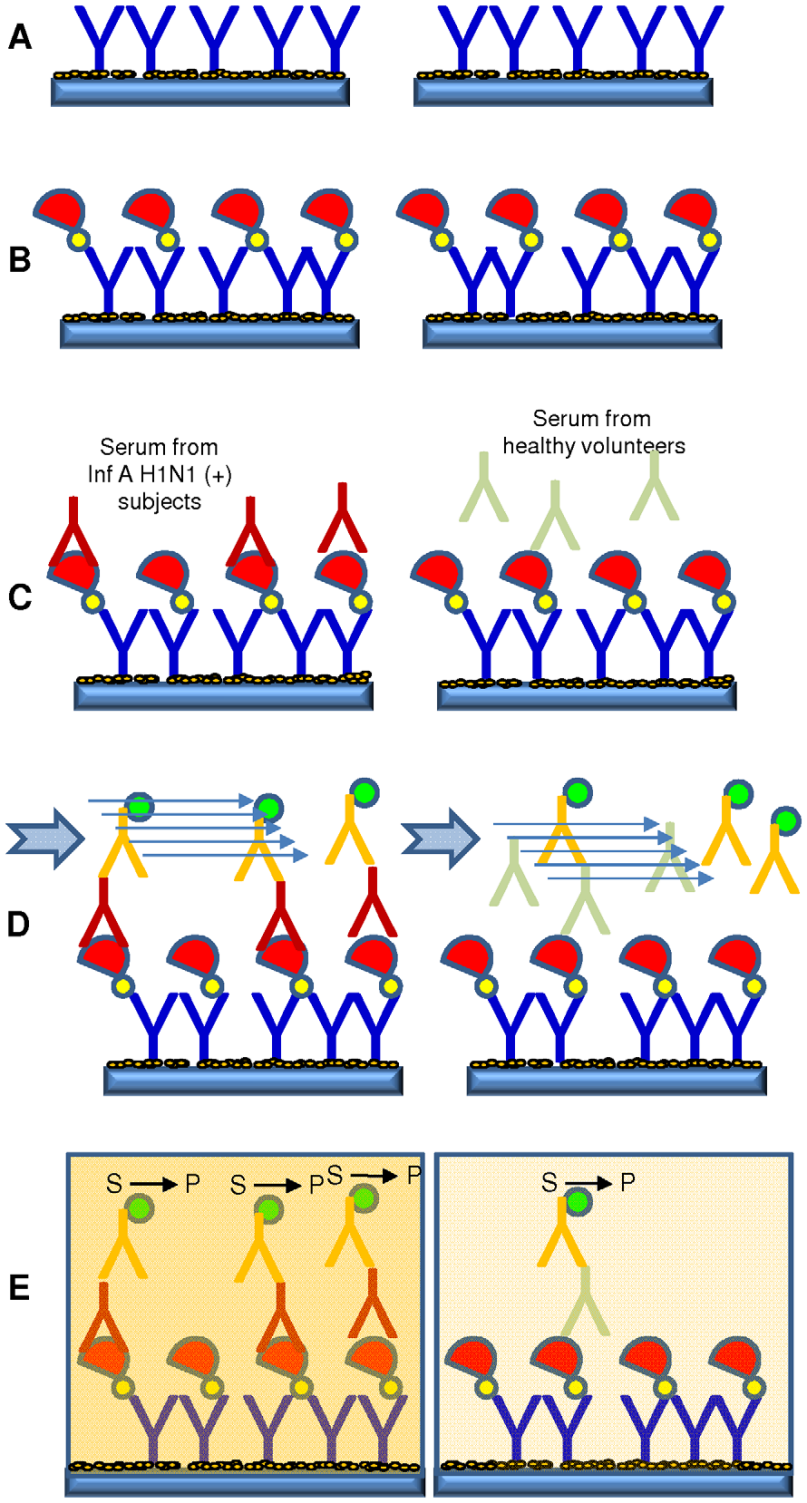
ELISA method designed to evaluate the relative concentration of specific antibodies (Y) anti-influenza A/H1N1/2009 virus in human serum and plasma. (A) Adsorption of anti-hystidine antibodies to the assay surface on 96-wells micro-assay plates and blockage of the remaining available surface with a commercial blocking solution. (B) Addition of the recombinant protein HA_50–274_-H1N1 (semi-circles). (C) Addition of serum samples potentially containing specific antibodies (Y) against the Influenza A H1N1/2009 virus. The left hand panel illustrates a scenario with a higher concentration of specific influenza antibodies. (D) Addition of a peroxidated anti-IgG human antibody (Y) to specifically bind the retained serum antibodies. (E) The addition of peroxidase substrate (S) enables the enzymatic reaction (S→P) with a proportional development of color.

We have successfully expressed a 25 kDa fragment of the globular region of the hemagglutinin of the 2009 H1N1 influenza virus (i.e. Influenza A/Mexico/InDRE4114/2009(H1N1)) (Figure 2) in the Rosseta-gami strain of *E. coli* [44], [45]. This protein, designated in our study as HA_50–274_-H1N1, ranges from residues 50 to 274 of the amino acid sequence of the hemagglutinin of the influenza 2009 H1N1 influenza virus. This is a highly conserved region (above 99% preserved) among the sequences of 2009 H1N1 influenza virus reported at NCBI. This protein conserves all antigenic sites previously reported for HA [29] and, as we demonstrate in the following section, specifically and selectively recognizes antibodies from serum samples of convalescent 2009 H1N1 influenza virus infected patients. We also added a sequence coding for a series of six histidines at the N-terminus of the protein (Figure 2) to facilitate downstream processes (this strategy allowed purification using Immobilized Metal Affinity Chromatography (IMAC)) and attachment to assay surfaces treated with anti-histidine antibodies (or alternatively Co^+2^ or Ni^+2^) (Figure 3a,3b).

### HA_50–274_-H1N1 protein preferentially recognizes antibodies from H1N1(+) patients

**Figure 4:**
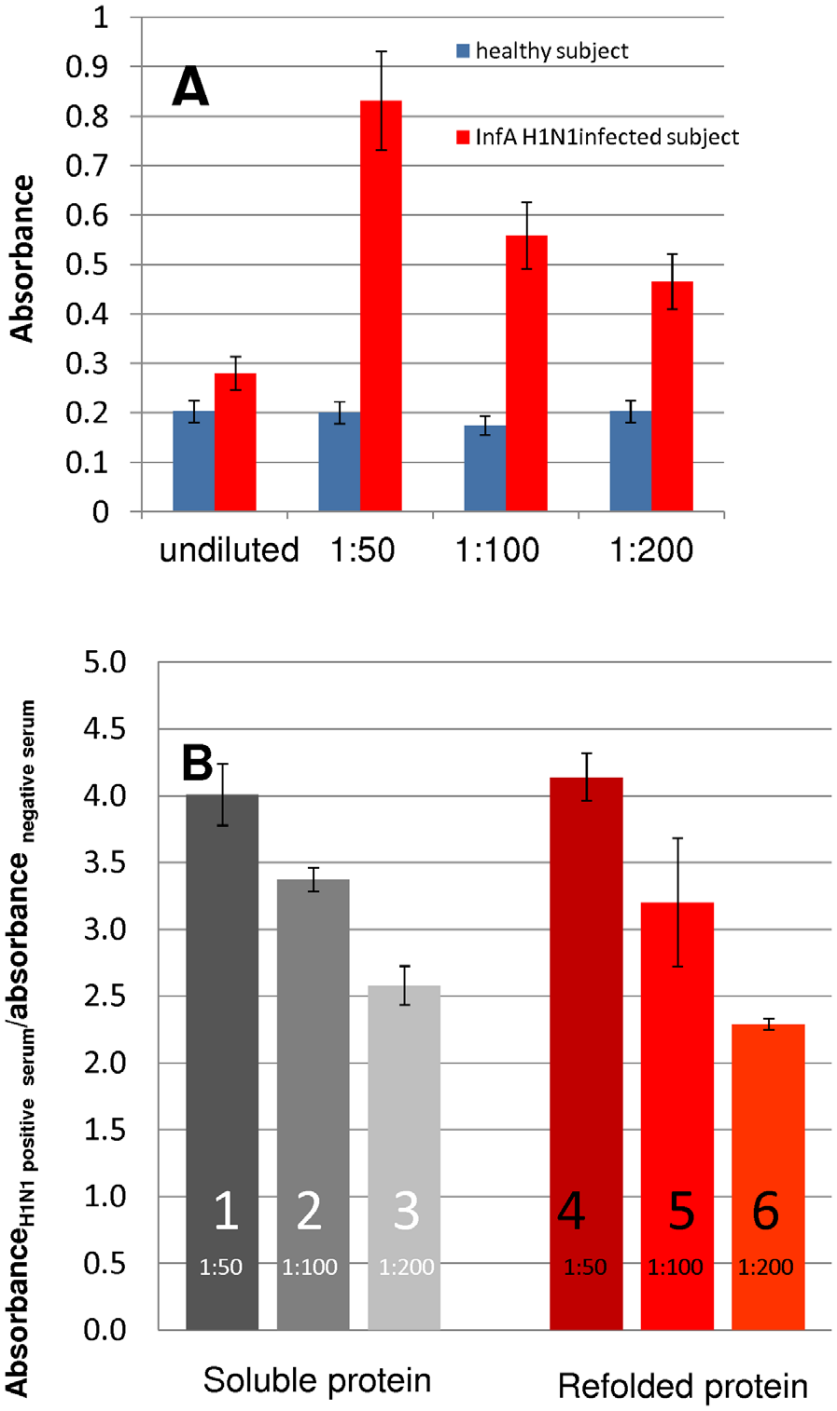
Serum from patients infected with Influenza A H1N1/2009 specifically recognize recombinant protein HA_50–274_-H1N1. (A) Results corresponding to undiluted serum samples and three different dilutions of serum in phosphate buffer saline solution (PBS) are presented (1∶50, 1∶100 and 1∶200). Blue bars correspond to absorbance signal from samples of healthy subjects. Red bars correspond to absorbance signal from samples of patients diagnosed as positive for Influenza A H1N1 taken three weeks after infection. Error bars were calculated based on the maximum percentage of variance (100*Average signal/standard deviation). (B) The ratio of the absorbance signal exhibited by a sample from a positive patient/absorbance signal of a sample from a healthy volunteer is presented. Results corresponding to three different serum dilutions (1∶50, 1∶100 and 1∶200) and two types of protein are presented (protein obtained directly in soluble form (columns identified as 1,2 and 3); and protein obtained after dissolution and refolding from inclusion bodies (columns identified as 4,5 and 6). Three replicates of each observation were considered. Error bars were calculated based on standard deviation.

Serum from patients infected with 2009 H1N1 influenza virus (as diagnosed using RT-PCR) specifically recognized the recombinant protein HA_50–274_-H1N1. Under certain conditions, this specific recognition could be used to conclusively discern serum samples from patients exposed and those from non-exposed subjects. In preliminary experiments, different ELISA strategies were tested in addition to the one detailed in the Materials and Methods section (see Figure 3). For example, direct binding of serum antibodies or protein HA_50–274_-H1N1 to the assay wells, as a first step of the assay, was examined. Based on our experimental observations, the method described here yields adequate reproducibility and a high signal/noise ratio. For adequate performance, dilution of serum samples was needed. In experiments comparing the absorbance signal in undiluted serum samples from infected and non-infected patients, a signal ratio (absorbance in samples from infected subjects/absorbance in healthy volunteers) of 1.39–1.59 was observed. When different dilutions (serum samples in PBS) were tested, in the range of 1∶50 to 1∶200, the signal ratio improved significantly (see Figure 4A).

For instance, typical signals in negative controls, using the anti-histidine antibodies and the HA_50–274_-H1N1 protein (with no serum addition), ranged between 0.050 and 0.070 absorbance units, with variance coefficients lower than 5%. Typical signals from serum samples of non-infected volunteers ranged between 0.15 and 0.40 absorbance units, with typical variance coefficients of less than 5% and standard deviations lower than 0.01 units. Typical signals of 2009 H1N1 influenza virus infected volunteers (as diagnosed by RT-PCR [27]) were at least 1.5 times higher.

We propose that diagnosis of exposure to the 2009 H1N1 influenza virus with this ELISA can be predicted on absorbance that is normalized to that of serum from uninfected subjects. Consequently, for reliable results, the inclusion of samples of non-exposed subjects (uninfected) subjects on every assay microplate is necessary. Therefore, for relative comparison among samples, absorbance values should be normalized based on the signal of one or several non-exposed subjects (uninfected subjects), and expressed as normalized absorbance (Abs_norm_), defined by equation 1:
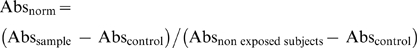
(1)where, Abs_sample_ is the absorbance signal of the sample, Abs_control_ is the absorbance signal of a negative serum albumin control, Abs_non exposed subjects_ is the average absorbance signal of samples from non-exposed subjects.

### Protein HA_50–274_-H1N1can be consistently produced

Recent experimental evidence confirms that the main antigenic sites in HA of the H1N1 influenza viruses are conformational [29], [37]. Therefore, proper folding of any recombinant influenza antigen is essential for adequate recognition. As an illustration, Chiu *et al.* documented the production of the HA1 domain of the hemagglutinin of the H5N1 influenza virus in *E. coli* cultures, its purification from inclusion bodies, and its proper solubilization, refolding and purification by affinity chromatography [43]. In their experiments, sera from convalescent animals challenged with H5N1 influenza virus were able to specifically bind recombinant fragments from HA. Remarkably, the activity of the recovered protein, exclusively measured in terms of specific recognition from infected rat serum antibodies, was strongly dependant on the refolding method [43].

**Figure 5:**
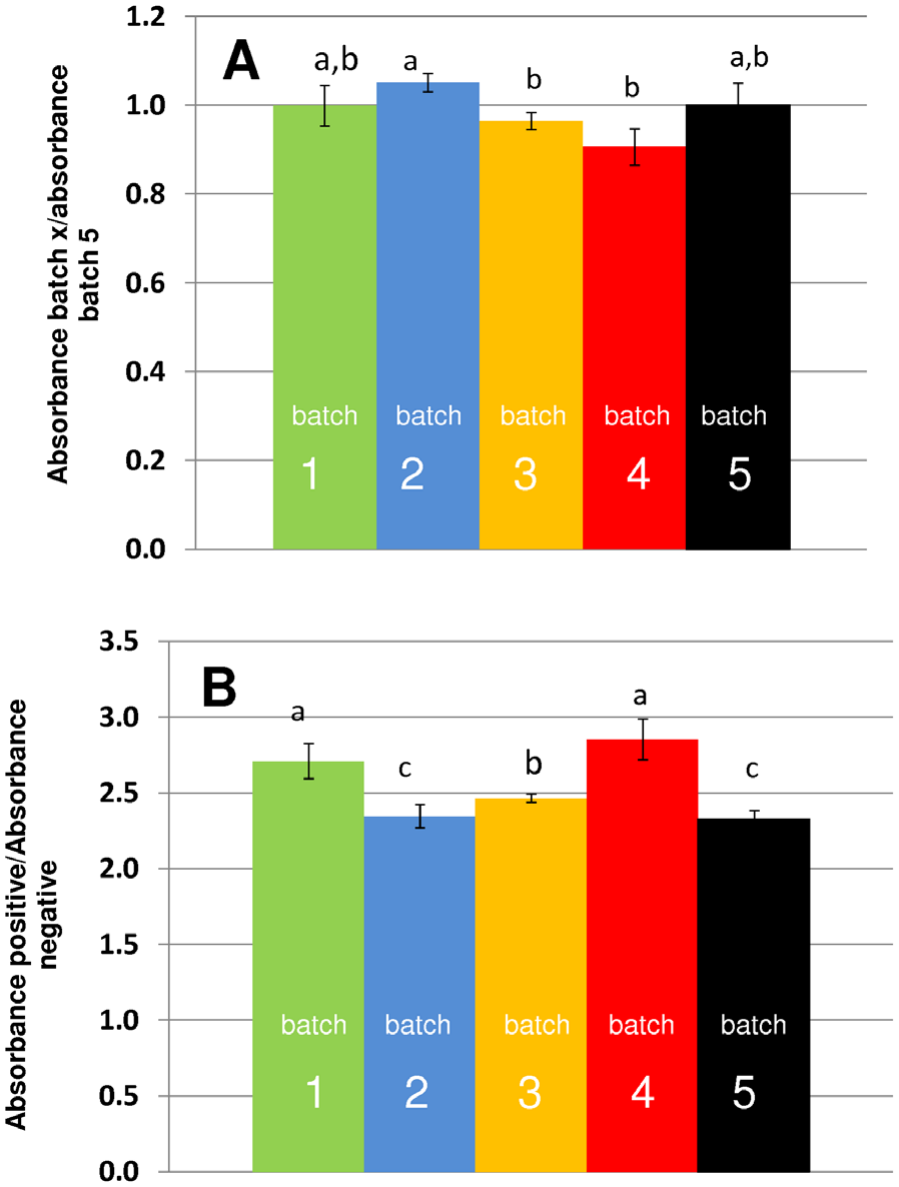
Indirect evaluation of proper refolding. (A) Biorecognition of antibodies from a positive patient observed for different production batches of protein HA_50–274_-H1N1. (B) Specific biorecognition ratio (ratio of biorecognition of antibodies from a positive patient serum and a negative subject serum) observed at different refolding batches derived from the same *E. coli* culture experiment. Variation among batches consisted in minor variations in the dissolution and refolding protocol used.

Correct folding, into a form resembling the native structure of the corresponding HA fragment of the 2009 H1N1 influenza virus, should be considered as crucial for adequate biological recognition. A series of experiments were therefore conducted to establish proper refolding of protein HA_50–274_ from inclusion bodies. For this purpose, a native soluble form of the HA_50–274_ protein was produced by expression in *E. coli* BL21 (DE3) pLysS variant C41, using a genetic construct that included a peptide signal for periplasmic expression. HA_50–274_ protein obtained by this method was taken as a reference of proper folding. Selective biorecognition of the native soluble and the refolded HA_50–274_-H1N1 protein by antibodies in serum from 2009 H1N1 influenza virus positive patients was established. Refolded HA_50–274_-H1N1 exhibited more than 90% selective bio-recognition with respect to native soluble HA_50–274_-H1N1 (Figure 4B) at different dilutions (1∶50, 1∶100 and 1∶200). This was consistently observed among different batches of the protein (A5a and 5B). Proper folding, defined here as the ratio of absorbance readings of serum from infected subjects obtained using originally soluble protein and refolded protein, was typically around 1 (+/−0.1) for all the production batches analyzed (see Figure 5A). Selective bio-recognition, defined as the ratio of absorbance signal between serum from a positive and a negative subject, was similar for different assays conducted with the same serum samples, regardless of the use of different protein production batches (Figure 5B). A more detailed documentation of the process of production and purification of protein HA_50–274_-H1N1 can be found elsewhere [45].

### Evolution of specific HA_50–274_-H1N1antibody titers after infection

**Figure 6:**
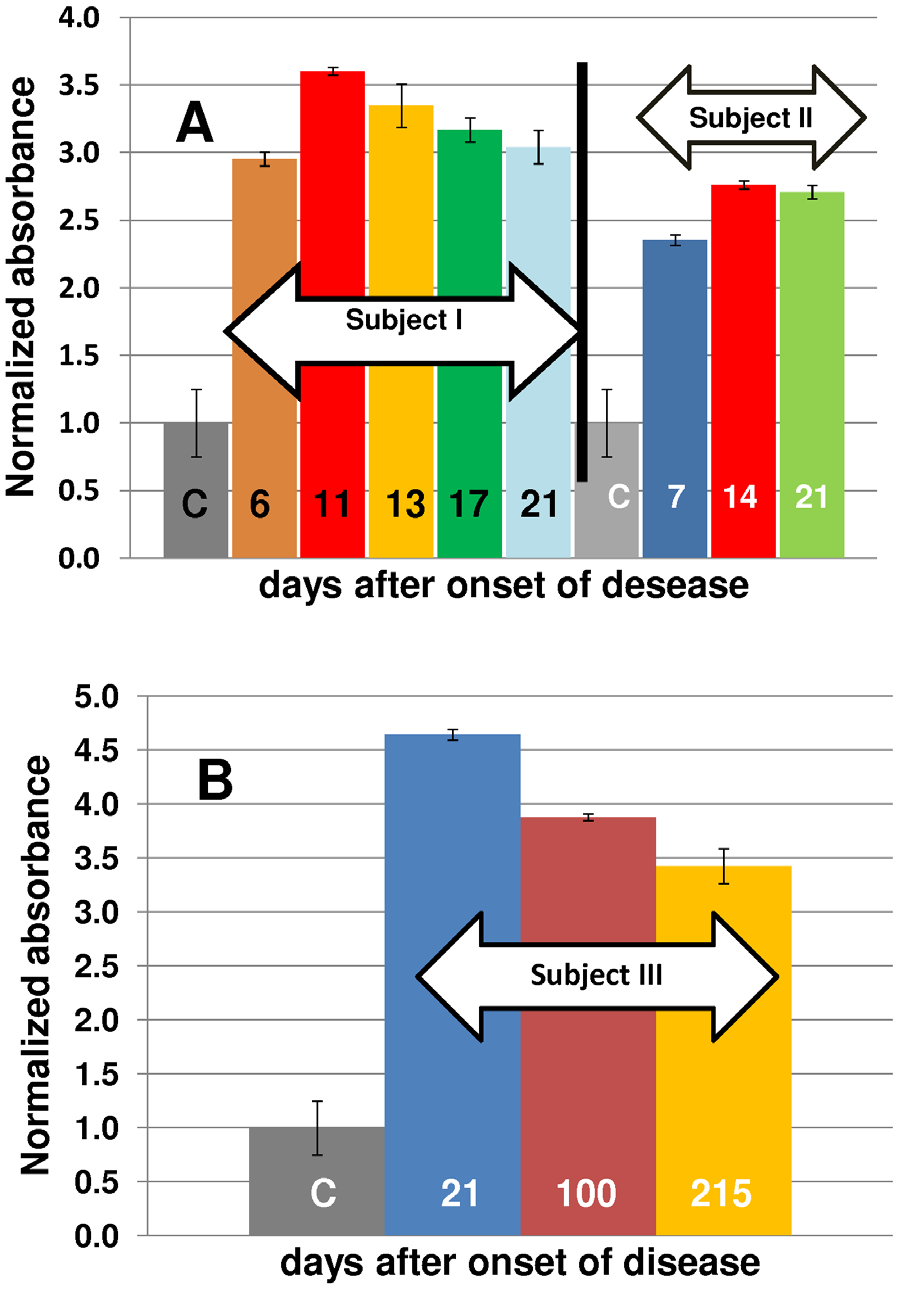
Evolution of the normalized absorbance signal of serum samples from patients diagnosed as positive to Influenza A/H1N1/2009. (A) Samples from two distinct patients were taken during the first three weeks after the onset of disease. Specific antibody titers more than double their basal value after day 7. (B) Samples from a patient taken at day 21, 100, and 215 after the onset of disease reveal that antibody titters remained high for at least seven months. Patients were diagnosed using RT-PCR protocols (WHO, 2009).

Significant antibody response to a new antigen in humans is normally considered to occur within two to three weeks of exposure. Some recent studies [15] have validated this for the specific case of influenza infection. Our results suggest that normalized absorbance values higher than two can be observed as soon as six or seven days after the onset of disease (Figure 6A). However, we consistently observed better discriminatory performance of the assay when performed with samples taken from patients three weeks after exposure to the 2009 H1N1 influenza virus.

The proposed immunoassay was used to follow the evolution of specific anti-influenza A/H1N1 titers in patients diagnosed as H1N1 positive. According to our observations, in most 2009 H1N1 influenza virus infected patients (4 out of 6 investigated cases) specific antibody titers fluctuated around a basal value during the first two week after infection. In these cases, two to three weeks after the positive diagnosis, the absorbance signal at least doubled its basal value. However, in 2 out of six cases, infected subjects doubled their basal absorbance signal as soon as six or seven days after onset of disease (Figure 6A). A similar pattern was observed in experiments where ferrets were immunized with protein HA_50–274_-H1N1 and monitored using the ELISA protocol described here (data not shown).

These results suggest that specific anti-2009 H1N1 influenza antibody titers could rise even during the first week after infection and in most cases will at least double their basal value by the third week after the onset of disease. Therefore, the immunoassay documented here is best suited for the analysis of antibody titers in samples from subjects exposed to the virus at least three weeks previously. Based on samples from a limited number of convalescent patients (5 cases), antibody titers appear to remain high at least seven months after infection (see Figure 6B). In general, a decrease in the normalized absorbance signal of less than 1.5 units was observed among convalescent patients followed from June 2009 to February 2010.

### Discrimination between infected and non-infected subjects

**Figure 7:**
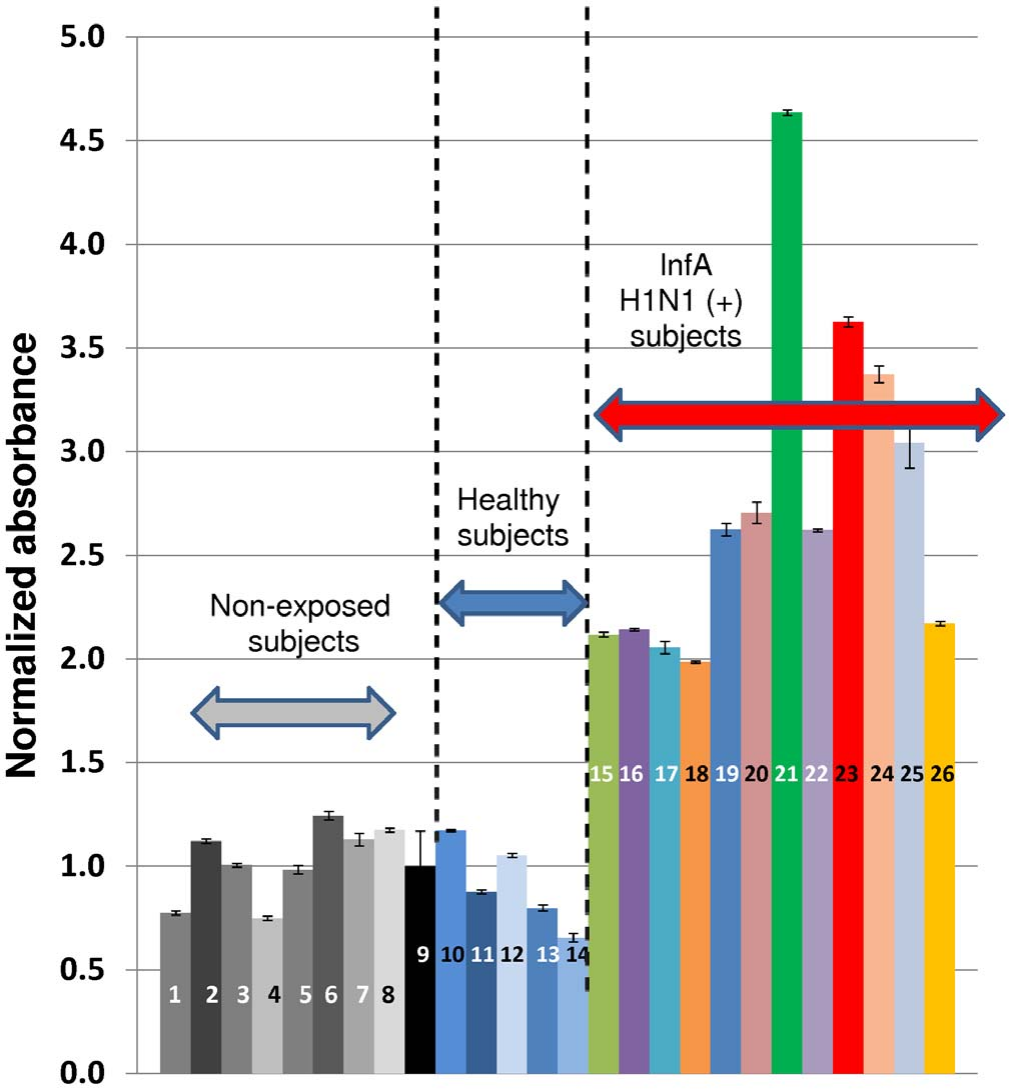
Serum from patients infected with Influenza A H1N1/2009 specifically recognize protein HA_50–274_-H1N1. Bars 1–8 (gray) correspond to absorbance signals from non-exposed subjects (samples taken from March to May 2008). Bar 9 (black) shows the average absorbance value of samples 1 to 8. Bars 10 to 14 (blue colors) correspond to absorbance signals from Inf A/H1N1 negative subjects. Bars 15–26 (different colors) correspond to absorbance signals from samples of Inf A H1N1 positive subjects (diagnosed two or three weeks before by RT-PCR). All signals were normalized with respect to the average absorbance signal observed in samples from non-exposed volunteers. Error bars form samples 1–8 and 10–26 represent one standard deviation based on at least three replicates on the assay in the same micro-plate experiment. Error bars form sample 9 represent one standard deviation based on all assays performed to samples from non-exposed volunteers.

Figure 7 shows results for the determination of the relative presence of 2009 H1N1 influenza virus antibodies in serum samples of volunteers, using the ELISA protocol described here. In all cases, 1∶50 serum dilutions in PBS were used. Results from three groups of volunteers are presented: (a) non exposed volunteers (serum samples taken from March to May 2008), (b) non infected subjects or 2009 H1N1 influenza-infected volunteers (one week after being diagnosed); and (c) patients diagnosed as positive to 2009 H1N1 influenza virus (at least three weeks after the most probable onset of disease).

Absorbance values were normalized based on the average signal of non-exposed subjects, and expressed as normalized absorbance (Abs_norm_), defined by equation 1.

**Figure 8:**
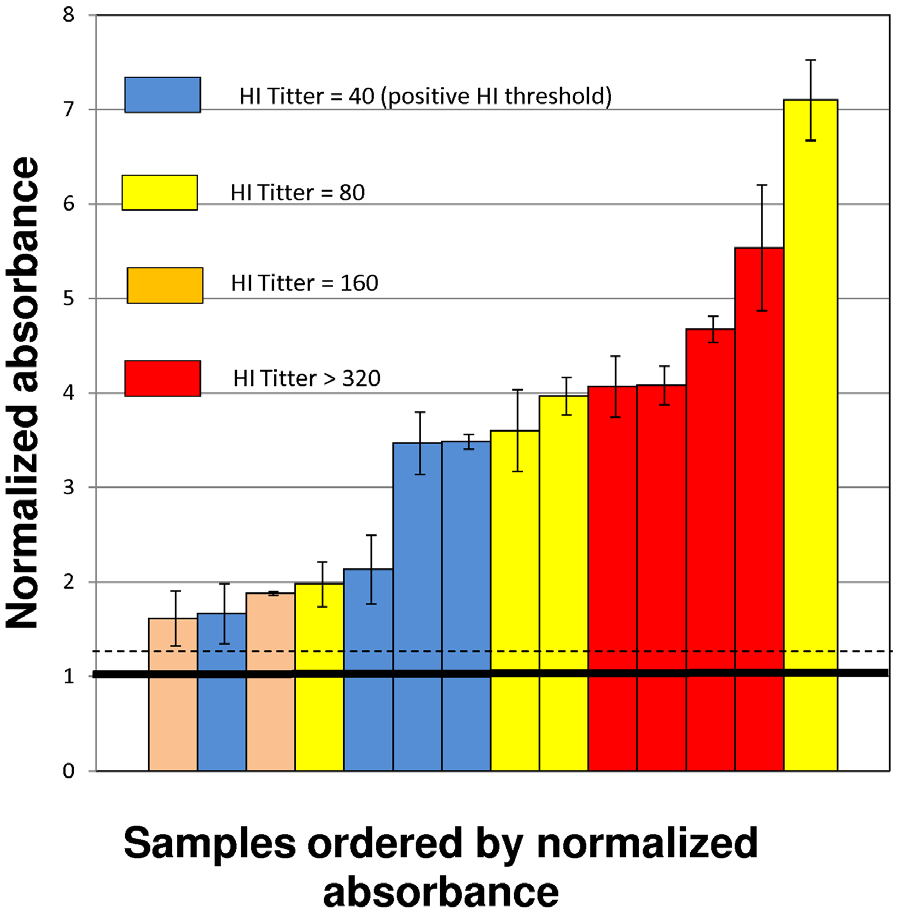
Validation of sensitivity against an HI assay. Normalized absorbance values for fourteen samples with positive anti H1N1/2009 titters based on an HI assay (samples that inhibited hemagglutination of turkey erythrocytes by the Ca/2009/H1N1 influenza virus strain at dilutions equal or higher to 1∶40). Colors indicate HI titter: HI titter = 40 (in blue); HI titter = 80 (in yellow); HI titter = 160 (in orange); HI titter>320 (in red). The proposed positive threshold for the ELISA method is indicated with a solid line (value = 1). One standard deviation is indicated with a dashed line (value = 1.25).

Samples from non-exposed subjects and negative subjects exhibited normalized absorbance values between 0.6 and 1.25. Variability in samples from negative subjects (and non exposed subjects) was relatively low, compared to absorbance values in samples with high specific serum antibodies titers. In the population tested, all serum samples corresponding to subjects within the third and fourth weeks after positive diagnosis exhibited absorbance values 2.0 to 4.5 times higher than samples from negative volunteers (Figure 7). Therefore, when normalized absorbance is used, the immunoassay appeared to clearly discern between serum samples from non-exposed subjects and samples from subjects exposed to the 2009 H1N1 influenza virus at least three to four weeks before. Based on our observations, a normalized absorbance value above 1.5 predicts exposure to the virus. Assay sensibility was further validated against results from Hemagglutination inhibition assays. Fifteen samples from subjects that exhibited positive titers in HI assays performed using the A/California/04/2009 virus were also analyzed by the described ELISA. Results are presented in Figure 8. All these samples exhibited normalized absorbance values higher than 1.5. In general, high HI titters (>320) were correlated with normalized absorbance values higher than 4.0.

### Assay variability

**Figure 9:**
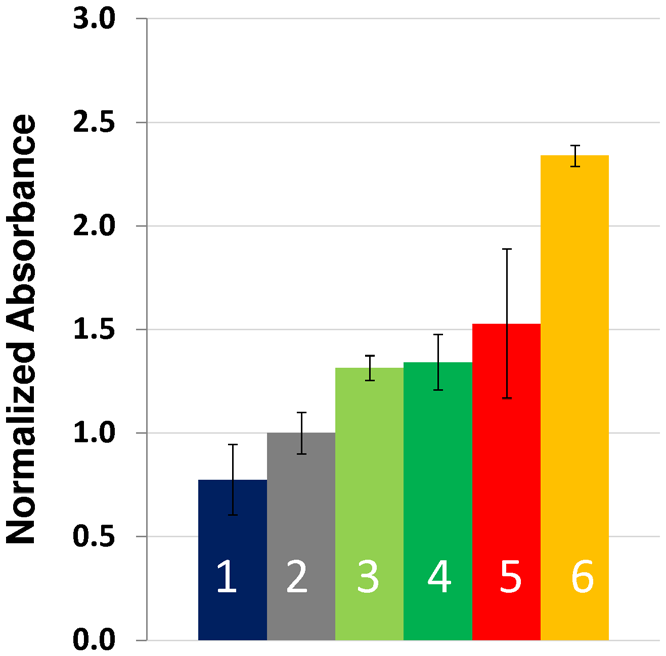
Reproducibility of the ELISA method for specific evaluation of anti Influenza A/H1N1/2009 antibodies in serum samples. Bars present the normalized absorbance value for samples of: three independent replicates of the assay on a sample of a non-exposed volunteer (bar 1); the average absorbance signal from eight different non-exposed volunteers (bar 2); three independent replicates of assays on samples taken two weeks after positive diagnostic on two different Influenza A/H1N1/2009 infected volunteers (bars 3 and 4); three independent replicates of assays on a sample taken three weeks after positive diagnostic on a Influenza A/H1N1/2009 infected volunteer (bar 5); three independent replicates of assays on a sample taken four weeks after positive diagnostic on a Influenza A/H1N1/2009 infected volunteer (bar 6).

The assay variability observed within the same ELISA experimental set was relatively low with respect to the absorbance signal, due to specific biorecognition. Error bars in Figure 6,7 and 8 indicate one standard deviation calculated from three repetitions of the ELISA runs in different wells within the same micro-assay plate. In order to characterize the assay variability among different ELISA experiments, a group of six serum samples from different volunteers and representative of different absorbance levels were selected. For each of these samples, assays were conducted in three different micro-assay plates with different reagent stocks. Each assay was carried out in triplicate. Each bar in Figure 9 represents the average of three experiment repetitions and at least nine assays in total (including replicates within each micro-plate). Error bars for samples 1,3,4,5, and 6 indicate the magnitude of one standard deviation, based on each set of at least nine experimental determinations. To establish the reproducibility and robustness of the assay among the whole spectrum of possible anti-2009 H1N1 influenza titters, the results presented in Figure 8 were selected to include cases with low, moderate, and high specific antibody titers.

From the five cases of samples presented, only one case exhibited important deviations (variability coefficients higher than 23%). In the rest of the cases, variability coefficients ranged between 5 and 15%. This variability did not compromise the ability of the method to discern between samples from exposed and non-exposed subjects. Bar 2 represents the average normalized absorbance and the standard deviation (error bars) associated with the analysis of eight serum samples from non-exposed subjects (samples taken from March to May 2008). The variability coefficient within samples from non-exposed subjects (therefore negative controls) was lower than 15%. Bar 1 corresponds to a sample from a healthy volunteer, presumably not exposed, whose normalized absorbance signal is statistically similar to that observed for non-exposed volunteers. The variability coefficient for this sample was determined as 20%. Bars 3 to 6 correspond to serum from subjects diagnosed as positive to 2009 H1N1 influenza virus between two and three weeks before the sample was taken. Statistically, the specific antibody titer exhibited by each one of these three samples is significantly higher than that determined for non-exposed subjects.

### Identification of exposed subjects within high risk populations

A feasible relevant application of the method described here is the identification of subjects who may be asymptomatic but who have indeed been exposed to the 2009 H1N1 influenza virus and therefore exhibit some level of specific antibodies. Presumably, these subjects would display at least partial protection against infection. Their identification would allow a more effective and rational vaccine administration in scenarios of limited supply.

**Figure 10:**
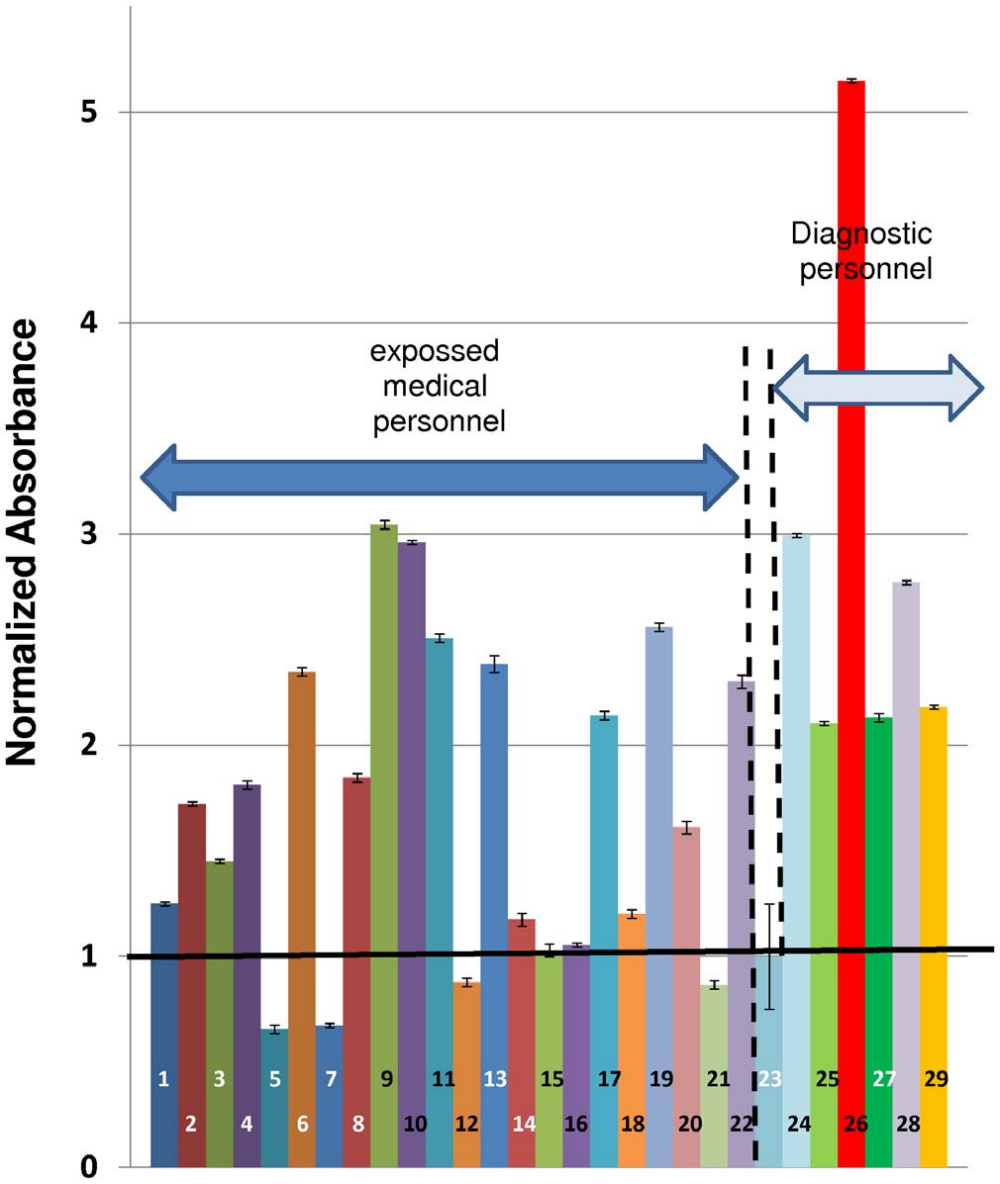
Normalized absorbance signals of serum samples from health care and diagnostic personnel in high exposure risk to the Influenza A/H1N1/2009 virus. Bars 1–22 present signals corresponding to asymptomatic health care workers. Bars 24–29 present signals corresponding to samples from H1N1 molecular diagnostic personnel. Bar 23 (negative reference) illustrates the average and standard deviation of eight samples from non-exposed subjects.

Serum samples from asymptomatic volunteers considered at high exposure risk (health workers or staff from an Influenza H1N1 diagnostic unit) were analyzed using the protocols described here. Of the 28 samples tested, 22 corresponded to medical personnel (medical doctors, nurses, and other health care workers) in close contact with patients diagnosed as infected with 2009 H1N1 influenza virus as confirmed by RT-PCR. The remaining 6 samples corresponded to operative personnel from our H1N1 molecular diagnostic unit at Centro de Biotecnología-FEMSA. All of these staff members participated directly in the manipulation of naso-pharyngeal samples from potentially infected patients and/or in the operations for extraction of viral RNA from these samples. Figure 10 shows results of the specific antibody titers, expressed in normalized absorbance units, found in these 28 samples.

Sixty percent of the samples from the medical personnel exhibited specific antibody titers higher than 1.5 normalized absorbance units (threshold observed to be reasonable to conclusively discern between exposed and non-exposed subjects). One hundred percent of samples corresponding to our molecular diagnostic unit staff showed high titers of anti-2009 H1N1 influenza virus antibodies. Although this set of 28 volunteers is a small and not necessarily representative sample, our results suggest that a high percentage of health care personnel who presumably come in contact with H1N1 patients could have a significant level of protective antibodies against the infection.

### Specificity

We have not conducted a formal study to evaluate possible cross-reactivity of protein HA_50–274_-H1N1 with antibodies directed against other Influenza A strains. However, some observations from the population of samples analyzed so far suggests that, as expected, cross-reactivity is minimal. Of all the volunteers identified by the assay as negative (26 individuals), 7 (27%) declared themselves to have had an influenza-like disease during the year 2008. Another 3 (11.5%) have been administered seasonal vaccines during 2008. Therefore, we would presume that at least 38% of the negative subjects should have circulating influenza A antibodies from seasonal strains.

Additional evidence of the specificity of the assay was provided by the analysis of samples from four volunteers diagnosed as positive and monitored throughout the first three weeks post-infection. All of these volunteers vouched to having had influenza-like illness recently (during the previous year). Two of these had received the 2008 seasonal influenza vaccine. In all four of these cases, their basal antibody titers were observed to be in the range of those typical of negative subjects (normalized absorbance value ≈1). In all cases, their antibody titers increased at least two fold by the third week after infection. These results suggest that possibly pre-existing antibodies against other influenza strains did not significantly interfere with the assay.

In conclusion, the typical HI strategy for evaluating viral infection suffers from a number of limitations that make this strategy unworkable in the face of pandemic infection. The requirement of vast quantities of fresh chicken or turkey erythrocytes as one of the main materials required for the assays, as well as the requirement for actual virus (and the corresponding need for equipment and manpower for large-scale viral culture), makes HI assays in typical laboratory settings impractical on a population scale. In the present work, we present a virus-free ELISA method that will allow the determination of relative concentrations of antibodies specifically directed against the 2009 H1N1 influenza virus. The immunoassay is based on the use of a recombinant fraction of hemagglutinin of the virus expressed in *E. coli*. The proposed method conclusively discerns between exposed and non-exposed subjects, particularly after the third week post-infection. Although validated in the context of 2009 H1N1 influenza virus, the same method can conceptually be extended to other seasonal and pandemic influenzas, simply by expression of the corresponding specific recombinant protein HA_50–274_.

In comparison with typically used methods to titer viral antibodies, this new method (a) does not require of the use of actual viral particles; (b) does not depend on the use of poultry erythrocytes; (c) it could be easily reproducible and implemented in any typical laboratory setting by personnel with typical laboratory skills.

Consequently, for massive epidemiological/clinical influenza studies, the protocols presented here would expedite the generation of reliable results that could be easily cloned in multiple laboratories. However, an independent validation of the ELISA specificity (i.e. a study in an independent test population validating a normalized absorbance threshold for specific diagnosis) will be necessary before wider application of this ELISA as a research tool can be recommended.

## Materials and Methods

### ELISA assay

We developed an ELISA method for the evaluation of presence of specific 2009 H1N1 influenza virus-antibodies in serum samples (Figure 3). Standard commercial 96-wells micro-assay plates (Corning®, Maxisorp™; USA) were used. One hundred µL of a solution of 5 µg/mL of mouse anti-histidine tag antibodies (AbD serotec®; UK) in PBS (pH 7.2+/−0.2) were dispensed per well. Plates were incubated for 12 to 16 h at room temperature to conveniently fix the anti-histidine antibodies to the bottom surface of each well. Excess antibody was removed by successive washing steps with PBS-Tween 0.05% and PBS. At least two cycles of 5 minutes with PBS-Tween 0.05% followed by two cycles of 5 minutes with PBS in an automatic micro-plate washing instrument are recommended. A volume of 300 µL of commercial blocking solution (Superblock® T20 PBS Blocking Buffer; Cat.No. 37516, Pierce®, USA) was added to each plate and incubated for at least 2 hours at room temperature to block the surface not covered with antibodies. Excess blocking solution was removed by successive washing steps with PBS-Tween 0.05%. A volume of 100 µL of a 10 µg/mL solution of a non-glycosylated histidine tagged recombinant protein was added to each well. The protein, the main component of the assay, consisted of a fragment of the hemagglutinin of the Influenza A/H1N1 virus (further details are given below). The protein solution was incubated for at least 1 h at room temperature to allow protein molecules to specifically bind to the anti-histidine antibodies through their histidine tags. Wells were washed repeatedly with PBS-Tween and PBS. At least two cycles of 5 minutes with PBS-Tween 0.05% followed by two cycles of 5 minutes with PBS in an automatic micro-plate washing instrument are recommended to remove unattached protein. A volume of 100 µL of the serum or plasma sample to be assayed was added to each well to test for specific bio-recognition. Four different serum dilution schemes in PBS were tested (1∶200, 1∶100, 1∶50, and undiluted samples). Best results were obtained at 1∶50 dilutions.

Serum samples were incubated at room temperature for one hour. After incubation, wells were washed repeatedly. At least two cycles of 5 minutes with PBS-Tween 0.05% followed by two cycles of 5 minutes with PBS in an automatic micro-plate washing instrument are recommended. To reveal the amount of antibody specifically bound in each well, a volume of 100 µL/well of an anti-human IgG antibody solution (1∶30000 dilution in PBS-Tween 0.05%) marked with horse radish peroxidase (Pierce®, USA) was used. After incubating for one hour at room temperature and washing repeatedly (at least two cycles of 5 minutes with PBS-Tween 0.05% followed by two cycles of 5 minutes with PBS in an automatic micro-plate washing instrument), a 100 µL volume of substrate solution (1-Step Ultra TMB-ELISA; Lot. 34028, Pierce®) was added to each well. After incubation for 15±5 min at room temperature in darkness, the enzymatic reaction was stopped by addition of 50 µL/well of a 1 M H_2_SO_4_. Color produced by the enzymatic reaction (from colorless to yellow) was evaluated by absorbance at 450 nm in a Biotek® microplate reader (USA).

### Recombinant hemagglutinin

Specifically, the protein used for the ELISA method described here was a fragment corresponding to a highly conserved region among sequences of 2009 H1N1 influenza viruses reported at NCBI. It was comprised of the amino acid sequence from residues 50 to 274 of the HA alignment consensus (referred as HA_50–274_-H1N1):

GVAPLHLGKCNIAGWILGNPECESLSTASSWSYIVETSSSDNGTCYPGDFIDYEELREQLSSVSSFERFEIFPKTSSWPNHDSNKGVTAACPHAGAKSFYKNLIWLVKKGNSYPKLSKSYINDKGKEVLVLWGIHHPSTSADQQSLYQNADAYVFVGSSRYSKKFKPEIAIRPKVRDQEGRMNYYWTLVEPGDKITFEATGNLVVPRYAFAMERNAGSGIIISD

The corresponding DNA sequence was obtained by back translation of the open reading frame and was optimized for *E.coli* expression (Figure 2). The gene was synthesized at DNA2.0 (San Diego, CA, USA) and cloned into a pJexpress404 vector (San Francisco, CA) in an *E. coli* Rosetta-gami® strain (strain deposited at ATCC by Alvarez *et al.* [44]). Given these genetic construction and culture conditions (aerobic culture in an instrumented 5 L bioreactor from New Brunswick Scientific, NJ, USA; 37°C, agitation at 500 RPM, pH 7, and induction using IPTG), practically all of the recombinant protein was expressed as insoluble inclusion bodies. After cultivation, biomass was centrifuged at 3000×g for 10 minutes. Twenty ml of TALON® xTractor Buffer (Clontech Laboratories, Mountain View, CA) were added per gram of wet cellular pellet to disrupt the cell membrane and extract the inclusion bodies. A concentrated solution of type I DNAases and Lysozyme 1X, was added to further degrade cell membranes and degrade DNA, thereby decreasing the viscosity of the solution and facilitating handling. The resulting solution was centrifuged at 12,000×g for 30 minutes at 4°C. A series of consecutive washing steps using PBS buffer solution rendered a precipitate containing the protein of interest in the insoluble fraction with a purity of above 90%. The protein was dissolved using 8 M urea solution and the resulting solution was passed through chromatography columns containing 2 mL of TALON® Metal Affinity Resin (Clontech Laboratories, Mountain View, CA) loaded with Co^+2^ ions and equilibrated at pH 8 or using a Ni^2+^ charged resin (Profinia Protein Purification System from Bio-Rad). While still attached to the resin through its histidine tags, the HA_50–274_ protein was treated with successive washes with PBS at pH 7 or 400 mM arginine at pH 8, to promote refolding. The protein was eluted with 150 mM imidazole at pH 7. Through this purification scheme, solutions in the range of 400 to 650 mg/L with purities exceeding 99.5% were obtained, as estimated by micro-elecrophoresis using an Experion® platform from Bio-rad (Hercules, CA). The robustness of this general purification strategy was evaluated by examining the performance of protein produced in five different refolding batches derived from the same *E. coli* culture experiment. Variation among batches consisted of minor deviations in the dissolution and refolding protocol previously described: 1) for batch 1, protein HA_50–274_-H1N1 was dissolved using a 8 M urea solution and refolded on-column with a 400 mM arginine in PBS using a Ni^2+^ charged resin (Profinia Protein Purification System from Bio-Rad); 2) for batch 2 a 8 M urea solution in PBS was used as solubilization agent and refolding occurred by direct dilution into 200 mM arginine using a dialysis system to exchange solvents; 3) for batch 3, an 8 M urea solution in PBS was used as solubilization agent and refolding occurred by direct dilution in a 400 mM arginine using a dialysis system to exchange solvents; 4) for batch 4 a 8 M urea solution in PBS was used as solubilization agent and refolding was conducted on-column by continuous passage of buffer solution PBS through a Ni^2+^ charged resin (Profinia Protein Purification System from Bio-Rad); and 5) batch five was the reference batch, where protein HA_50–274_-H1N1 was expressed in its soluble form using a signal peptide for periplasm translocation (see [46]).

### Serum samples

This study was conducted according to the principles expressed in the Declaration of Helsinki. The study was approved by the Institutional Review Board of the Escuela de Biotecnología y Salud del Tecnológico de Monterrey. All volunteers provided written informed consent for the collection of samples and subsequent analysis.

Different experiments with human serum samples were conducted. In a first experiment, aimed to validate the ability of the ELISA protocol to discriminate subjects with high H1N1 specific antibody titers in general population, serum samples from 25 volunteers were collected and analyzed using the ELISA protocols presented here. Samples were diluted 1∶50 in PBS. These 25 samples corresponded to 3 different groups. Eight samples were collected in Monterrey Nuevo León, México, from March to May 2008, practically one year before the onset of the pandemic. Therefore, these subjects most probably corresponded to volunteers with no exposure to the Influenza A/H1N1/2009 virus. Five samples were taken from healthy volunteers, selected on the basis of a questionnaire designed to exclude any persons with symptoms possibly associated with influenza like illness within a period of six months previous to this study. In addition, 11 samples corresponded to volunteers diagnosed as positive to Influenza A/H1N1/2009 two to four weeks before the sample was taken (presumably three to five weeks after infection). Positive volunteers were recruited from regular patients from the Hospital San José del Tecnológico de Monterrey admitted within the period of April to October 2009. All diagnoses were confirmed using the specific RT-PCR protocol developed by the Center for Prevention and Disease Control (CDC) in Atlanta, Georgia, USA, and recommended by the World Health Organization (WHO) [24] as the reference method for the detection of the human Influenza A/H1N1/2009 virus.

In a second experiment, 14 samples corresponding to subjects with HI titters equal or higher than 40 (conventionally considered as exposed to the virus based on the HI assay) were tested by ELISA. A Influenza A/H1N1/CA/04/2009 virus strain and Turkey erythrocytes were used for the HI experiments. Dilutions in the range of 1∶40 to 1∶320 were considered. In a third experiment, serum samples from 28 subjects assumed to be at high exposure risk to the Influenza A/H1N1/2009 virus (health care workers and diagnostics personnel) were studied. All of these subjects declared not to have been clinically diagnosed or symptomatic for influenza-like illness during at least during the year 2009. These volunteers were diagnosed as negative to Influenza A or Influenza A/H1N1 using the specific RT-PCR protocol developed by CDC (Atlanta, USA) and recommended by WHO for Human Influenza A/H1N1/2009 [27]. Volunteers were selected from health workers who directly attended H1N1 positive patients at Hospital San José (Monterrey, México) and personnel directly involved in the operation of the molecular diagnostics lab at a Diagnostic Center in Monterrey located at the Centro de Biotecnología-FEMSA at Tecnológico de Monterrey.

In addition, the evolution of specific titers against the Influenza A/H1N1/2009 virus was followed in four volunteers diagnosed as positive using RT-PCR protocols. Subjects were required to donate blood samples during three weeks from the day they were diagnosed. Samples from one of these volunteers corresponding to 21, 100, and 250 days after the onset of disease were collected and analyzed to determine long term evolution of specific antibody titters.
